# Advanced Foam Dressing of Modified Graphene Nanoparticles Loaded in Bacterial Cellulose/Calcium Alginate Matrix

**DOI:** 10.1021/acsomega.5c00609

**Published:** 2025-06-30

**Authors:** Thamyres Freire da Silva, Jacilane Ximenes Mesquita, Erika Patricia Chagas Gomes Luz, Alexandre Lopes Andrade, Henry Kobs, Edson Holanda Teixeira, Antônio Gomes de Souza Filho, Andreia Fonseca de Faria, Adriano Lincoln Albuquerque Mattos, Fábia Karine Andrade, Rodrigo Silveira Vieira

**Affiliations:** a Adsorption Separations Research Group, Department of Chemical Engineering, 28121 Federal University of Ceará, Fortaleza, Ceará 60455-760, Brazil; b Integrated Biomolecules Laboratory, Department of Pathology and Forensic Medicine, 28121 Federal University of Ceará, Fortaleza- Ceará 60430350, Brazil; c Department of Physics, 28121 Federal University of Ceará, Fortaleza - Ceará 60440-900, Brazil; d Department of Environmental Engineering Sciences, 3463 University of Florida, Gainesville, Florida 32611, United States; e Embrapa Tropical Agroindustry, Fortaleza, Ceará 60511-110, Brazil

## Abstract

Patients dealing with chronic wounds frequently experience persistent pain and a heightened risk of infection due to microbial contamination. To improve the quality of life for these patients while reducing treatment duration and costs, advanced wound dressings are being developed. This study aimed to develop a wound dressing from natural bacterial-derived cellulose fibers (BC) and calcium alginate (A), which were functionalized with silver-containing graphene oxide nanoparticles (GOAg). Carbon-based nanomaterials, such as GOAg, are recognized for their antimicrobial properties, noncytotoxic nature, and their strong ability to absorb exudate, which is crucial for controlling infections in dermal lesions. In vitro testing revealed that the BC-A-GOAg dressing possessed optimized characteristics, including a uniform distribution of GOAg nanoparticles within the polymeric blend of BC and A. When in direct contact with bacterial cells in suspension, the BC-A-GOAg dressing exhibited 74% antimicrobial activity against *Staphylococcus aureus* and 59% against *Pseudomonas aeruginosa* . Additionally, the antimicrobial BC-A-GOAg demonstrated no significant cytotoxicity to mouse fibroblast cells (L-929), maintaining 90.8% ± 5.2% cell viability after 48 h of exposure. Furthermore, the in vitro assessments showed that the BC-A-GOAg dressing could inhibit the activity of the myeloperoxidase enzyme, highlighting its effectiveness in reducing inflammation.

## Graphical abstract

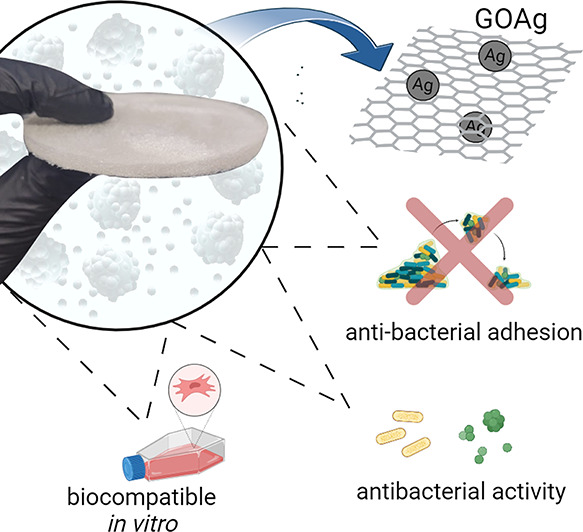

## 1 Introduction

A wound is characterized by an impairment in the integrity of the skin or mucous membrane and can arise from intentional, traumatic, or ischemic origins. Chronic wounds, which persist beyond 6 weeks and sometimes endure for multiple years, significantly impact various aspects of an individual’s quality of life.^1^ This can lead to damage to self-esteem due to associated disabilities, including pain, infectious symptoms, inflammation, and compromised sleep quality, all of which disrupt overall well-being.^2,3^ The implications of these factors extend to various aspects of personal life, self-perception, and the individual’s societal and familial roles.^4^

The following characteristics are common to all dressings: impermeability to water and other fluids, allowing gas exchange; easy application and removal without causing trauma; aiding in hemostasis to prevent bleeding; protecting against mechanical trauma and infection; limiting movement of tissues around the ulcer; absorbing secretions; and relieving pain.^5^ Local treatment of wounds has three fundamental objectives based on its pathophysiology and behavior: treating infection, removing necrotic tissue from the wound bed, and managing excess exudate.^6^ Chronic wounds can be treated using dressings, as well as topical and systemic antibiotics to prevent infections. However, it is essential to combat the indiscriminate use of systemic or local antibiotics to prevent bacterial resistance, which could render treatment with the same antibiotics unfeasible in the future.

Bacterial cellulose (BC) is a commonly used polysaccharide in fabricating wound dressings and hydrogels due to its biocompatibility and potential to interact with therapeutic agents, thereby facilitating cutaneous healing.^7–9^ Sodium alginate is a natural polymer widely used in dressings due to its comparatively affordable prices, biocompatibility, and high exudate absorption capacity.^10–12^

Graphene oxide (GO) is a nanosheet material comprising sp^2^ carbon interspersed with hydroxyl, carboxyl, carbonyl, epoxide, phenol, lactone, and quinone groups, which promotes chemical reactivity.^13,14^ In biomedical contexts, GO has been shown to enhance drug delivery through its increased surface area and antimicrobial characteristics.^15,16^ The GO possesses surface characteristics, particularly the high density of oxygenated functional groups that enable effective hydrogen bonding with cellulose.^17–19^ Its extensive surface area also contributes to enhanced compatibility within polymeric matrices and facilitates interactions with bacterial cells. Additionally, GO exhibits excellent stability and dispersion in aqueous media,^20^ which simplifies the integration with hydrophilic biopolymers such as cellulose.^19^ Enhancing the antibacterial potential of GO for contact can be achieved by incorporating silver nanoparticles (AgNPs), creating a dual bactericidal effect, where GO interacts through its sheets, while released Ag^+^ ions cause membrane damage upon bacterial entry.^21,22^ The silver incorporated into the GO interacted with sulfur and phosphorus rich cellular components enhances bactericidal efficacy.^23^ Previous work has demonstrated the potential of AgNPs incorporated into the BC polymer, showing antimicrobial action against Gram-positive and Gram-negative strains.^24–26^

Luz et al. developed a bacterial cellulose (BC) and GOAg membrane by immersing BC (in its natural form) in a GOAg suspension.^27^ The authors reported significant surface adsorption of the GOAg nanocomposite, resulting in fast release of silver and subsequent loss of antimicrobial activity. This present work proposes the production of a membrane with GOAg in a more homogeneous and distributed fashion throughout the BC/calcium alginate foam matrix, creating an antimicrobial membrane for wound dressing applications. The nanoparticles were produced using the Turkevich method and modified for AgNPs with GO to prevent them from clumping together.^28,29^ A sponge composed of deconstructed cellulose (BC), calcium alginate (A), and GOAg was developed through the drying of the hydrogel and the cross-linking of sodium alginate with calcium chloride (CaCl_2_). This process resulted in the formation of a water-resistant membrane made of calcium alginate. The spongy structure created a membrane that adsorbed significantly more water than what was reported by Luz et al.^27^ In this study, BC-A-GOAg demonstrated a precisely controlled, point-by-point release of silver over 3 days, resulting in slower and more sustained release of the silver, which extended its antimicrobial activity. We also created BC-A and BC-A-GO controls to compare the antimicrobial effectiveness of the GOAg composite in the BC-A-GOAg membrane and to identify any differences in physicochemical properties.

## 2 Materials and Methods

### 2.1 Materials

Sodium carbonate (Na_2_CO_3_), potassium carbonate (K_2_CO_3_), potassium bromide (KBr), Dulbecco’s Modified Eagle Medium and mitomycin C were obtained from Sigma-Aldrich LTDA. Sodium chloride (NaCl), potassium chloride (KCl), dibasic sodium phosphate (Na_2_HPO_4_) and potassium phosphate monobasic (KH_2_PO_4_) from Dinâmica Química Contemporânea LTDA were used to PBS solution. Ethanol (C_2_H_6_O), calcium chloride (CaCl_2_), sodium bicarbonate (NaHCO_3_) and sodium alginate were bought from Dinâmica Química Contemporânea LTDA. Glucose, peptone, citric acid monohydrate yeast extract, anhydrous dibasic sodium phosphate, carboxymethylcellulose sodium salt (CMC) and potassium carbonate from Neon were used to produce membranes. Silver nitrate (AgNO_3_), SYNTH, sodium citrate (Na_3_C_6_H_5_O_7_), Vetec, and graphene oxide purchased from Cheap Tubes were used in the nanoparticle synthesis reaction. Brain Heart Infusion Broth (BHI) (K25–1400) and Mueller-Hinton medium (MHB) (K25–1400) from Kasvi. All reagents were of analytical grade and used without further purification.

### 2.2 Methods

#### 2.2.1 BC Production and Purification

Bacterial cellulose (BC) was produced by static fermentation of *Komagataeibacter hansenii* (ATCC 53582) in HS (Hestrin and Schramm) medium^30^ following Vasconcelos and collaborators.^31^ The bacteria and culture medium were removed entirely, and purified BC was washed with an alkaline solution under heating. The BC was immersed in distilled water (DI) at 80 °C for 1 h, and the liquid was discarded; this procedure was repeated. The membranes were immersed in a 0.3 mol L^–1^ potassium carbonate solution at 80 °C for 1 h. The solution was discarded, and the process was repeated. The excess base was removed by successive washes with distilled water until neutralization (pH 7.0). The membranes were stored in a Scotch bottle at 4 °C until use.

#### 2.2.2 Preparation of GOAg Nanocomposite

The reaction to produce silver-anchored graphene nanoparticles (GOAg) was based on the methods described by Luz et al. and Turkevich et al.^27,28^ The GO was initially dispersed in DI at a concentration of 312.5 μg mL^–1^. This dispersion was achieved utilizing a tip ultrasonicator (Hielscher, UP100H) for a duration of 30 min operating at a frequency of 50% within a 0.5 cycle, and conducted under cooling conditions in an ice bath. Subsequently, a silver nitrate solution at a concentration of 420 μg mL^–1^ was incorporated into the dispersion. The resultant mixture was maintained under magnetic stirring at 1100 rpm for 30 min while ensuring it remained shielded from light and continued to be cooled in the ice bath. Following this, the mixture underwent further sonication using the same parameters for an additional 5 min while stirring at 500 rpm. A reaction system was then established comprising a round-bottomed flask, which was heated to 130 °C and was also subjected to stirring at 500 rpm, with a condenser in place for cooling purposes. Upon reaching boiling point, a sodium citrate solution at a concentration of 250 μg mL^–1^ was gradually added to the flask, and the mixture was maintained under heat for a period. The Raman spectroscopy (WITec alpha300, with an Andor optical system and coherent anti-Stokes scattering) was conducted to identify characteristic bands of graphene and silver doped into the sheets. The thermogravimetric analysis (TG) of GO and GOAg (5 mg) was conducted using a simultaneous thermal analyzer (STA) (STA 6000, PerkinElmer, Massachusetts, USA). The samples were heated from 20 to 700 °C at a rate of 10 °C/min under a synthetic air atmosphere composed of 20% O_2_ and 80% N_2_, with a flow rate of 20 mL/min. The TGA data were processed using the OriginPro 8 software (OriginLab Corp., Northampton, Massachusetts, USA). For the Fourier transform infrared spectroscopy (FTIR), the GO and GOAg samples were mixed with KBr at a ratio of 3% (m/m). The mixture was then pressed into tablets under a pressure of 3 tons. The FTIR analysis was performed using a PerkinElmer Spectrum.

#### 2.2.3 Foam Dressing Production

The process of producing the functionalized membranes is illustrated in Figure 1. The foams were produced from a hydrogel base consisting of bacterial cellulose (BC) and sodium alginate (A), forming BC-A, hydrogel with GO nanoparticles (BC-A-GO), and hydrogel with GOAg nanoparticles (BC-A-GOAg).

**Figure 1:**
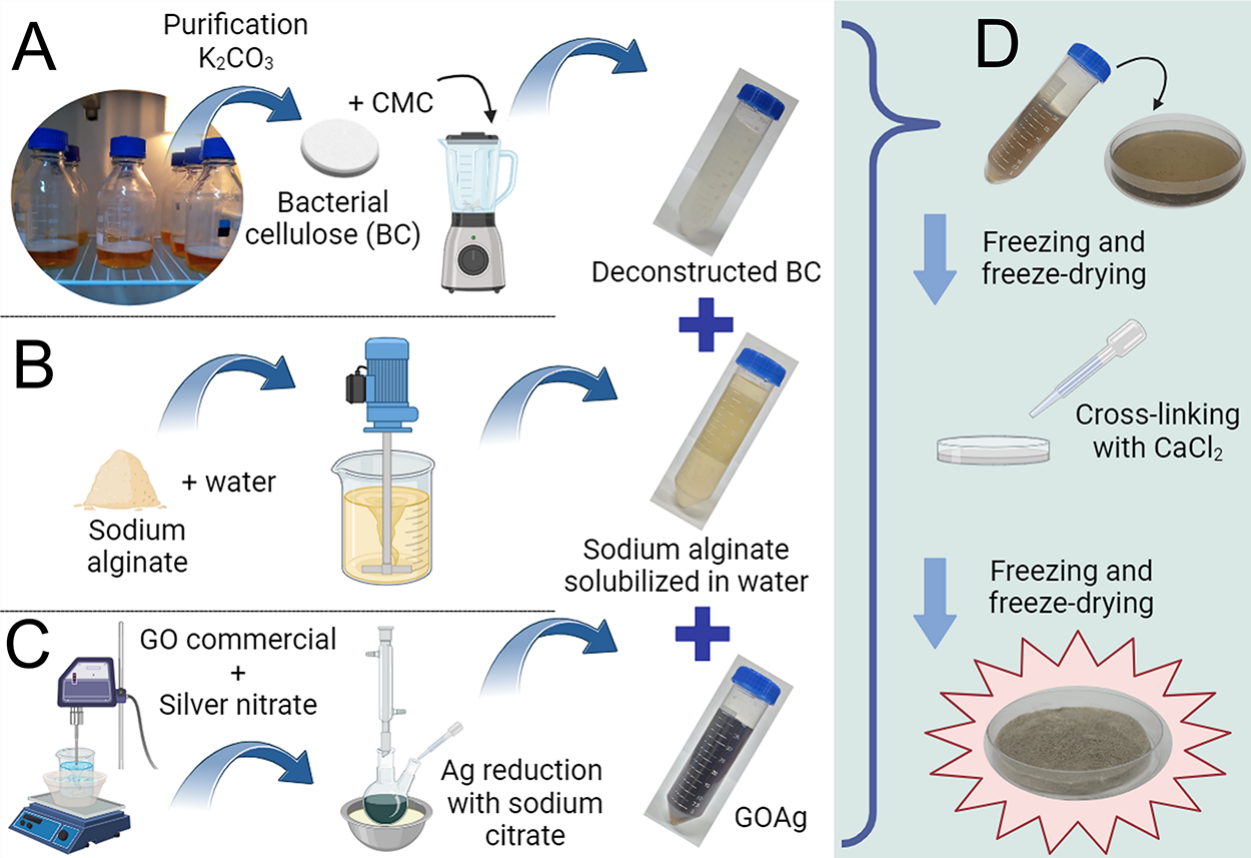
BC production by static fermentation, deconstruction of BC in a liquefier, and addition of CMC to stabilize the BC gel (A). Solubilization of sodium alginate in water by stirring at room temperature (B). Production of GOAg by GO dispersion using ultrasound and silver reduction using the Turkevich method (C). Production of the GO-A-GOAg sponge involves mixing gels and nanoparticles, followed by freezing, drying, cross-linking with CaCl_2_, and processing in a freeze-dryer (D). Created in BioRender. Fonseca de Faria, A. (2025) https://BioRender.com/lqek893.

The dressing was produced following an adapted method from Kirdponpattara et al.^32^ This involved mixing 20 mL of deconstructed bacterial cellulose hydrogel (2% w/v) stabilized with CMC (0.5%, w/v CMC in H_2_O) and sodium alginate (2%, w/v alginate per hydrogel) combined with 10 mL of either GO or GOAg dispersion, totalizing 30 mL of hydrogel with 41.6 μg mL^–1^ GO and 56 μg mL^–1^ Ag anchored to GO. The mixture was spread on Petri dishes (90 × 15 mm), frozen at −20 °C, and then dried. A solution of calcium chloride (0.5 mol L^–1^) was added dropwise, acting as a cross-linking agent for the sodium alginate. After cross-linking, the dressings were thoroughly washed thrice with 50 mL of distilled water each to remove any excess cross-linking agent. The dressings were then frozen again at −20 °C and freeze-dried. Finally, the membranes were sterilized using an autoclave at 121 °C for 15 min at 1 atm.

#### 2.2.4 Physicochemical Characterization of the Functionalized Membranes

The morphological characteristics of the BC-A, BC-A-GO, and BC-A-GOAg membranes were acquired through a Scanning Electron Microscope (SEM) (Quanta 450 FEG – FEI). Chemical characteristics of the same membranes were obtained using a PerkinElmer Spectrum Two coupled to a Pike Technologies ATR/MIRacle. The absorbance readings of the ZnSe crystal ranged from 4000 to 800 wavelengths with a resolution of 4 cm^–1^. Thermogravimetric analysis (TGA) was conducted to investigate the thermal stability of the of the BC-A, BC-A-GO, and BC-A-GOAg membranes (model STA 6000, PerkinElmer, Massachusetts, USA). The temperature was swept from 20 to 700 °C at a heating rate of 10 °C/min under a synthetic air atmosphere of 20% O_2_ and 80% N_2_ at a flow rate of 20 mL/min. The *in vitro* release of silver was performed using Franz diffusion cells,^33,34^ which consist of donor and receptor compartments with an effective diffusion area of 1.8 cm^2^. The donor chamber was sealed with parafilm to prevent evaporation. A nylon screen with 150 μm openings (Sefar Filtration, USA) was placed beneath the film for mechanical support. The receiving chamber was filled with buffer solution, serving as the release medium to ensure optimal dissipation conditions. The experiments were carried out at 37 °C with agitation at 100 rpm. The medium in the receiving chamber was routinely replaced with an equivalent volume of fresh buffer. The concentration of silver ions released was measured using atomic absorption spectrometry (AAS). A calibration curve was established in advance using a silver standard within a concentration range of 0.1 to 20 ppm. The degree of swelling was determined following the methodology established by Liu and collaborators.^35^ The dressings produced were immersed in distilled water to observe their swelling behavior in this solvent, in triplicate. After each immersion, the excess water was removed using a filter paper and weighed at intervals of 0, 1, 3, 5, 7, 10, 20, 30, 40, 50, and 60 min at 25 °C. The degree of swelling was calculated as the percentage of mass gain relative to the initial mass, as described in eq 1.

$$\mathrm{DS}=\left(W_{s}-W_{d}\right)/W_{d}$$ 1

Where DS is the degree of swelling, Ws is the mass of the sample after immersion, and Wd is the mass of the dry sample before immersion. All measurements were carried out in triplicate. X-ray diffraction (XRD) patterns of the membrane samples were recorded using a PANalytical X’Pert PRO (Netherlands). The results were acquired in continuous scanning mode with a step size of 0.013° (2θ). Monochromatic Co–Kα1 radiation was utilized, operating at 40 kV and 40 mA. The mechanical testing procedure adhered to the ASTM D 638–99 standard.^36^ Samples were cut into strips measuring 12 × 60 mm, with 12 replicates prepared and stored at 23 °C in a 50% humidity environment for 48 h. Each sample was individually measured for height, width, and thickness. The uniaxial tensile test was conducted using the DL-3000 apparatus from EMIC, with a crosshead displacement rate of 12.5 mm/min and a 100 N load cell (S10). A Student’s *t* test was employed to analyze the mechanical parameters, with significance determined at a p-value of less than 0.05.

#### 2.2.5 Antibacterial Activity

Minimum inhibitory concentration (MIC) tests were conducted on GO and GOAg in suspension. The BC-A, BC-A-GO, and BC-A-GOAg membranes were evaluated for their direct antimicrobial activity after 4 h, as well as for bacterial adhesion and indirect antimicrobial activity of 10-day extracts of the dressings. The experiments using bacteria were conducted in quadruplicate.

##### 2.2.5.1 Minimum Inhibitory Concentration (MIC) of GO and GOAg

This assay used *Staphylococcus aureus* (ATCC 25923) and *Pseudomonas aeruginosa* (ATCC 27853) as model microorganisms. The minimal inhibitory concentration (MIC) of GO and GOAg was determined using the microdilution method, performed in 96-well polystyrene plates. This procedure was standardized under the M7-A 10th edition of the ″Methods for Dilution Antimicrobial Susceptibility Tests for Bacteria That Grow Aerobically,″ which is a guideline developed by the Clinical and Laboratory Standards Institute (CLSI).^37^ The plates were subsequently analyzed using a microplate reader at a wavelength of 620 nm.

##### 2.2.5.2 Antimicrobial Activity for Direct Method

The protocol was adapted from de Faria et al. to evaluate the dressing’s direct antimicrobial activity in contact with suspension microorganisms.^38^ *Staphylococcus aureus* (ATCC 25923) and *Pseudomonas aeruginosa* (ATCC 27853) were used in the test. The cells were activated in brain heart infusion broth (BHI) and incubated for 24 h at 37 °C. Following incubation, the microorganism suspension was centrifuged at 4 °C at 9000 rpm for 5 min. The supernatant was discarded, and the cells were resuspended in a saline solution (0.9%) to reach a cell concentration equivalent to 10^6^ CFU mL^–1^. A 0.9% (w/v) saline solution (30 mL) was poured into sterilized Falcon tubes. A saline control containing bacteria was carried out to assess the normal state of the cells. Additionally, a control of the dressing in contact with the saline solution was conducted to evaluate the effectiveness of the sterilization process and to verify the presence or absence of live microorganisms. Three membrane coupons measuring 4 cm^2^ were placed in the same tube, and the triplicate was reproduced. After 3 h of contact (37 °C) between the coupons and the solution, 10 μL of the suspension was diluted (10-fold, in 0.9% saline solution) and plated in BHI agar. These plates were incubated for 12 h at 37 °C, after which the cell colonies were counted. The values were expressed as a percentage and compared with the control.

##### 2.2.5.3 Evaluation of the Antiadhesion Properties of Membranes

The antiadhesion properties of the membranes were determined using a protocol adapted from Faria et al. and *S. aureus* and *P. aeruginosa* as model microorganisms.^38^ The microorganisms were activated in BHI and incubated for 24 h at 37 °C. After incubation, they were centrifuged at 4 °C for 5 min at 9000 rpm and then resuspended in a 0.9% saline solution to achieve a concentration of 10^6^ CFU mL^–1^. Four cm^2^ coupon samples were placed in contact with a 30 mL suspension of microorganisms in triplicate at 37 °C for 3 h. Following the incubation period, the samples were washed with 2 mL 0.9% saline solution to remove nonadhered cells. The membranes were transferred to a new tube containing 10 mL of saline solution and sonicated for 20 min to detach the adhered cells. The resulting sonicated suspension, prepared in triplicate at each dilution (10-fold dilutions ranging from 10^–1^ to 10^–6^ in 0.9% saline), was then dripped (10 μL) onto Petri dishes containing BHI agar. These plates were incubated for 12 h at 37 °C, after which the cell colonies were counted. The results were expressed as a percentage and compared to the control.

##### 2.2.5.4 Indirect Antimicrobial Activity

The indirect antimicrobial activity was evaluated using extracts of BC-A, BC-A-GO, BC-A-GOAg, and the commercial dressing *Aquacel Ag Extra*. One cm^2^ of each membrane type was placed in 1 mL sterile pH 7.4 PBS containing 10^6^ CFU mL^–1.^ As described in a previous study,^39^ these samples were incubated for 3 days at 37 °C to release the active ingredients, producing membrane extracts. Then, 100 μL of the sample and 100 μL of the microorganism suspension were placed in 96-well polystyrene plates with U-shaped bottoms. After 18 h of incubation, bacterial growth was evaluated by measuring absorbance at 620 nm using an automated microplate reader (SpectraMax i3x, Molecular Devices, Sunnyvale, USA). The graph was plotted by expressing the data as a percentage relative to the control group of bacteria with normal growth in the culture medium, according to eq 2.

$$\mathrm{Inhibition}\;\mathrm{of}\;\mathrm{microorganisms}\;(\%)=\left(\frac{F_{sample}-F}{F_{control}}\right)\times 100$$ 2

#### 2.2.6 Cytotoxicity Test

The cytotoxicity assay was conducted using fibroblasts from the L-929 cell line, following the guidelines outlined.^40^ The membranes were placed in contact with a cell culture medium to obtain an extract, according to ISO 10993–12.^41^ The tests were conducted in quadruplicate, with three repetitions (n = 3) between different cell passages to a new culture bottle. The cells were seeded in 96-well plates, and the extract was then added to the wells. The extract was kept in contact with the cells for 24 and 48 h at 37 °C. A solution of resazurin (25 mg mL^–1^) was added to the wells. The metabolization of resazurin was assessed by measuring fluorescence at an excitation wavelength of 560 nm and an emission of 590 nm, which allowed for the calculation of cell viability. Cell viability was calculated according to eq 3.

$$\mathrm{Cell}\;\mathrm{viability}\;(\%)=\left(\frac{F_{sample}-F}{F_{control}}\right)\times 100$$ 3

The fluorescence is respectively: *F*_*sample*_, with the sample in contact with cells, *F* with extract without cells, and *F*_*control*_ with cells in contact with DMEM supplemented with 10% (v/v) fetal bovine serum (FBS) and containing 1% (v/v) penicillin-streptomycin antibiotic.

#### 2.2.7 Fibroblast Migration

An aliquot of 1 mL of L-929 cell suspension, containing 2.5 × 10^5^ cells mL^–1^, was added to each well in a 24-well plate. The plate was incubated for 24 h at 37 °C in an atmosphere of 5% CO_2_ and 95% humidity to allow for cell adhesion and growth until 100% confluence was achieved. After 24 h, the supernatant was removed and Mitomycin C (10 μg mL^–1^) was added to inhibit cell mitosis. A total of 1 mL of Mitomycin C solution, prepared in DMEM, was added to the plate, which was then incubated at 37 °C for 2 h. The cytostatic agent solution was removed and washed with PBS buffer. Next, an artificial lesion (scratch) was created using a sterile 200 μL plastic tip, made in a vertical direction, radial to the center of the bottom of each well. The cells were then gently washed again with PBS pH 7.4 to remove detached cells. After removing the PBS, 1 mL of extract from the BC, BC-A-GO, and BC-A-GOAg samples was added, following the protocol outlined by Silva et al.^42^ The analysis was performed in duplicate for each sample, along with a control using only the culture medium. Using a guideline marked on the bottom of the plate, photographs were taken of the same area with a digital camera attached to an inverted microscope (Nikon) at 0, 6, 12, 18, and 24 h after the addition of the extract. The scratch area from the photographs taken at the specific times was determined using ImageJ, and the migration rate was calculated using eq 4.^43^

$$\mathrm{Migration}\;\mathrm{rate}\;(\%)=(\frac{\mathrm{Scratch}\;\mathrm{dis}\tan\mathrm{ce}\;(0\;h)-\mathrm{Scratch}\;\mathrm{dis}\tan\mathrm{ce}\;(N\;h)}{\mathrm{Scratch}\;\mathrm{dis}\tan\mathrm{ce}\;(0\;h)})\times 100$$ 4

#### 2.2.8 Inflammation Test by Myeloperoxidase (MPO) Enzyme Activity

Activated or dying neutrophils release myeloperoxidase (MPO), a protein capable of inducing an inflammatory response. Blood samples from healthy individuals was collected in tubes containing EDTA. We followed the *Cayman-*adapted Neutrophil Myeloperoxidase Activity Assay Kit protocol for the experiments. The neutrophil isolation procedure began by transferring 5 mL of the collected blood into a tube, followed by the addition of an equal volume of Cell-Based Assay Buffer. To separate the blood cells according to density, histopaque from a kit was added to the tube. The blood was diluted and then centrifuged at 500 g for 30 min at 25 °C and the clear yellow supernatant was discarded. A Cell-Based Assay Red Blood Cell Lysis Buffer was added to the neutrophil layer, which was vortexed and left to stand for 10 to 15 min. The tube was then centrifuged at 1200 rpm for 10 min to separate the lysed red blood cells. The supernatant was discarded, and the neutrophils were resuspended in RPMI containing 1% BSA, followed by another centrifugation at 1500 rpm for 5 min. This step was repeated to further remove cell debris. Aliquots of 100 μL of the cells at a concentration of 1 × 10 ^5^ cells/100 μL were added to conical-bottomed microtubes and treated with membrane extract for 4 h. A positive control from the kit was used with human leukocyte polymorphonucleocytes, neutrophils with an inhibitor, and PMA (phorbol-12-myristate-13-acetate) to stimulate the release of MPO from the neutrophils. Tetramethylbenzidine (TMB) was then added to check the reaction with MPO, which indicated an increase in blue color. The increase was measured by absorbance at 650 nm and recorded 1 to 10 min after the first reagent was added. The enzyme activity calculated using eq 5.

$$\Delta A650=\left[\frac{A650(\mathrm{Time}5\min)-A650(\mathrm{Time}1\min)}{4\min}\right]$$ 5

To obtain the dressing membranes extract, 1 cm^2^ pieces were placed in contact with PBS for 10 days. They were evaluated for induction of inflammation on the first day at 2, 4, 6, 8, and 10 min and on the 10th day at the same times mentioned above. The tests were conducted in duplicate to calculate the mean and statistical standard deviation.

#### 2.2.9 Statistical Analysis

The results obtained were initially subjected to descriptive analysis and normality determination. The statistical analysis was performed using GraphPad Prism 8.0 software. Given that the samples demonstrated a normal distribution, a two-way Analysis of Variance (ANOVA) was employed for inflammatory analyses and water uptake ability. At the same time, one-way ANOVA was utilized for the other analyses. P values lower than 0.05 were considered statistically significant. Symbol Meaning according the software: ns for *P* > 0.05; * for *p* ≤ 0.05; ** for *p* ≤ 0.01; *** for *p* ≤ 0.001 and **** for *p* ≤ 0.0001.

## 3 Results and Discussion

### 3.1 Physicochemical Characteristics of GO, GOAg, and the Functionalized Membranes

Figure 2A displays the dispersion of GO and GOAg in water, along with the BC-A-GO and BC-A-GOAg membranes. The Raman spectra reveal the characteristic D (1335 cm^–1^), G (1589 cm^–1^), and 2D (1671 cm^–1^) bands associated with GO, as well as peaks indicative of Ag^+^ ions (983 and 454 cm^–1^), as shown in Figure S1, Supporting Information. The SEM images of the membranes revealed the presence of large pores in both BC-A-GO and BC-A-GOAg (Figures 2 B-E). Additionally, the BC-A-GO and BC-A-GOAg displayed numerous overlapping porous sheets. Evaluating thermal stability is essential for assessing mass loss and ensuring temperature resistance during autoclave sterilization, which is commonly employed for biomedical materials.^44^ The TG analyses were conducted to investigate the effect of sterilizing the GO, GOAg, and membrane solutions through autoclaving at 121 °C, as well as the impact of residual silver on the degradation of the BC and alginate membranes (Figure 2F). A mass loss of approximately 15% was observed across all three samples during the initial thermal event up to 100 °C, which is typically associated with water loss or membrane degradation. Notably, GOAg exhibited less mass loss compared to GO alone. At 500 °C, GO and GOAg retained a mass of 35.86% and 62.00%, respectively, indicating that residual silver was retained up to that temperature. The BC-A, BC-A-GO, and BC-A-GOAg membranes displayed similar profiles, maintaining masses of 82.66%, 77.29%, and 78.40%, respectively, Figure 2F. Therefore, while autoclaving may be suitable for dressing sterilization while preserving material properties, it is recommended that future studies consider alternative sterilization methods such as ethylene oxide or γ radiation. Although sodium alginate begins to thermally degrade at around 180 °C, the interactions with BC and graphene composites were explored to determine whether the degradation would occur in a similar or different temperature range.^45^ The BC membrane began to degrade at approximately 280 °C, thereby providing the polymer matrix with enhanced thermal stability.^46,47^ GO can exhibit a mass loss of up to 21% at 200 °C, as previously reported.^48^ The BC-A, BC-A-GO, and BC-A-GOAg samples exhibited similar vibrational bands, as illustrated in Figure 2G. The presence of calcium alginate is indicated by the elongation of the 1601 and 1429 cm^–1^ bands, which appear asymmetrically. Similarly, BC-A-GOAg exhibited comparable vibrational bands (Figure 2G). The presence of calcium alginate can be identified by the asymmetrical elongation of the vibrational bands at 1601 and 1429 cm^–1^. Jiang et al.^49^ noted that purified BC displays these bands with less intensity, as emphasized by Vasconcelos et al.^31^ Moreira Filho et al., highlight that only alginate displays the characteristic bands corresponding to O–H (3348 cm^–1^), symmetrical bending (1336 cm^–1^), symmetrical stretching of C–H/-CH_2_ (2892 cm^–1^), as well as bending (1422 cm^–1^, 1370 cm^–1^, and 895 cm^–1^), and asymmetrical stretching of C–O–C (1170 cm^–1^).^50^ The characteristic bands of the aromatic rings of the GO and GOAg sheets appear superimposed on that of the matrix at around 1600 cm^–1^. In Figure 2H, the XRD peaks for BC-A were observed at the angles of 16.2° and 26° for the control BC-A. These peaks are characteristic of bacterial cellulose, as previously demonstrated by Kirdponpattara et al.^32^ According to the literature, calcium alginate shows a more amorphous structure, without peaks.^51^ The XDR peaks corresponding to the AgNPs anchored at the GOAg sheets in the BC-A-GOAg membrane appear at 31°, 39.5°, 44.5°, 60.1°, 64.6°, and 68.5°. In their data, Forouzandehdel et al. showed silver-related peaks at 57.1° and 67.2°.^52^ The study of Torabi et al. displayed peaks at 38.20°, 44.56°, and 64.60°, corresponding to the (111), (200), and (220) planes of crystallized silver with face-centered cubic structure.^53,54^ The peaks at 31° and 60° may be attributed to cross-linked impurities on the membrane surface.^55,56^

**Figure 2:**
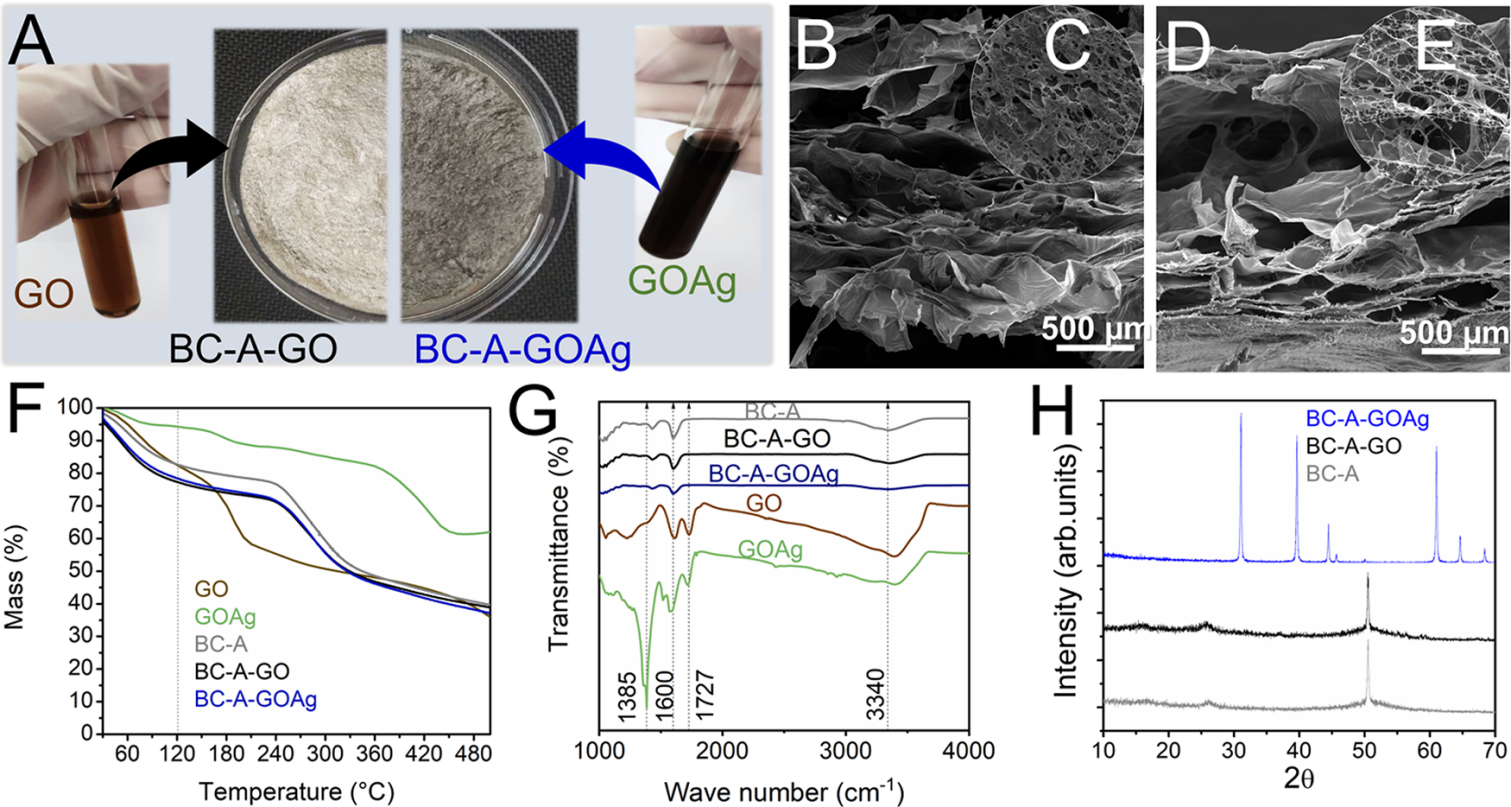
Digital photographs of the GO and GOAg solution and BC-A-GO and BC-A-GOAg membranes (A). The transversal SEM micrographs of BC-A-GO (B) and BC-A-GOAg (D) show irregular porosity. SEM micrographs of superficial BC-A-GO (C) and BC-A-GOAg (E). Physicochemical characterization of the GO nanosheets, nanocomposite GOAg, and the functionalized membranes (BC-A, BC-A-GO, and BC-A-GOAg) (F, G). Thermogravimetric analysis (F), FTIR (G), and XDR (H) for the samples.

In terms of water absorption, both BC-A and BC-A-GO exhibited. In terms of water absorption, both BC-A and BC-A-GO exhibited comparable absorption profiles for the initial 60 min, absorbing 21.38 and 22.30 times their dry weight values, respectively (Figure 3A). In contrast, BC-A-GOAg demonstrated a reduced water absorption profile, capturing only 15.70 times its weight. The water absorption capacity of BC-A-GOAg was enhanced through the sponge-forming procedure, achieving an absorption rate of up to 15.70 times its dry weight. In contrast, Luz et al. reported a significantly lower water absorption of 0.9 times its dry weight for BC/GO-Ag, indicating lower swelling in relation to the values found for BC in the literature.^27^ Vasconcelos et al. studied the BC polymer matrix used by Luz et al. in physiological saline solution (PS), and only for BC showed swelling of 1.5 times its dry weight.^57^ In this study, the swelling ability of BC-A-GOAg was lower than that reported by Kirdponpattara et al.,^32^ who observed absorption of 48 times its dry weight. This discrepancy may be due to Kirdponpattara et al. using a higher cellulose to sodium alginate ratio of 70:30,^32^ whereas this study used a lower ratio of 50:50.

**Figure 3:**
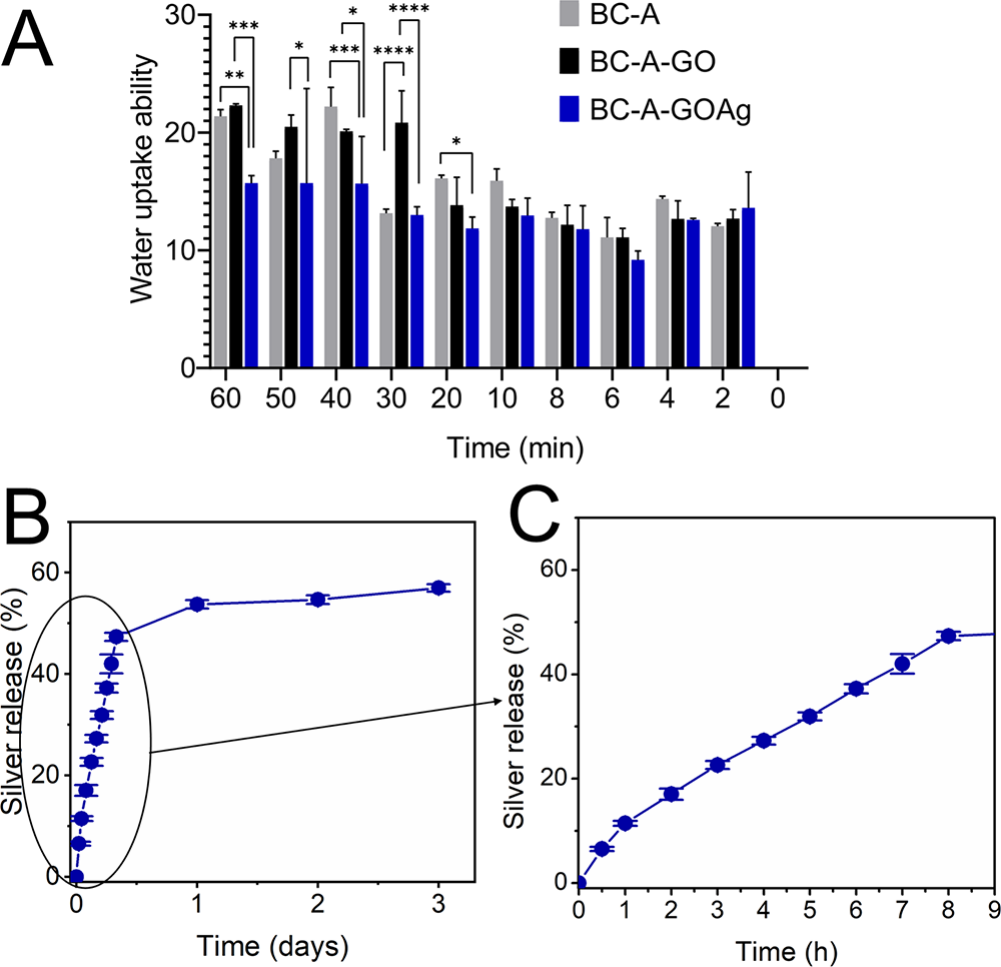
Water uptake ability of BC-A, BC-A-GO, and BC-A-GOAg membrane dressings over 1h (A), accumulated silver released from BC-A-GOAg membrane up to 3 days (B), and until 8 h quantified by AAS (C).

The BC-A-GOAg dressing incorporates silver nanoparticle technology, along with GO, at a concentration of 33 μg cm^–2^. Data shows that the dressing releases a maximum cumulative concentration of 18.8 μg cm^–2^, accounting for 57% of the total silver content (Figure 3B). Notably, within the first hour, 11.45% of free silver is released, and with prolonged contact with PBS, the concentration of silver ions steadily increases, reaching 47.34% in 8 h (Figure 3C) and 53.74% in 24 h, as confirmed by AAS. This sustained release of silver offers significant advantages, including a reduction in dressing change frequency to minimize patient discomfort and delivering a robust and lasting antimicrobial effect.^58^

The BC-A-GO and BC-A-GOAg hydrogels exhibit a lower resistance profile compared to the pure BC membrane, which has a tensile strength of 1.1 ± 0.05 MPa, an elongation at break of 26.6 ± 1.28%, and a Young’s modulus of 5.3 ± 0.79 MPa.^31^ This difference arises because, during the hydrogel production process, the natural BC fibers are broken down, while the membrane structure is preserved through the cross-linking of sodium alginate with calcium chloride, resulting in the formation of calcium alginate. The presence of GO-Ag sheets enhances the interaction between the nanomaterial and the polymer matrix, leading to a more robust structure. Previous studies on the mechanical strength of BC, alginate, and AgNPs have indicated a final tensile stress of 0.05 ± 0.06 MPa for the BC/alginate membrane and 0.17 ± 0.05 MPa for the BC/alginate/AgNPs composite.^59^ Therefore, the presence of GO-Ag strengthens the polymeric framework of the BC-A-GOAg dressing (Table 1).

**Table 1:** Mechanical Properties of the Control and Functionalized Dressings^a^

sample	tensile strength (MPa)	elongation at break (%)	Young’s modulus (MPa)
BC-A-GO	0.18 ± 0.12 c	1.46 ± 0.62 a	14.79 ± 10.21
BC-A-GOAg	0.69 ± 0.45 a	1.26 ± 0.42 b	58.24 ± 33.78

a Values are expressed as mean ± standard deviation (*n* = 8) and show nonstatistical difference (α = 0.05) by Tukey test.

### 3.2 Antibacterial Activity

A minimum inhibitory concentration (MIC) analysis was conducted, as shown in Figure 4, to evaluate the antimicrobial activity of GOAg. Dilutions of the GOAg suspension were made and exposed to *Staphylococcus aureus* and *Pseudomonas aeruginosa* cells. Successive dilutions were prepared from the initial concentrations of 97.65 μg mL^–1^ of GO and 131.25 μg mL^–1^ of AgNO_3_ using a 2-fold serial dilution method. The results indicated significant antimicrobial activity, with inhibition levels exceeding 75.4% for *S. aureus* and 79.1% for *P. aeruginosa* up to the sixth dilution of GOAg.

**Figure 4:**
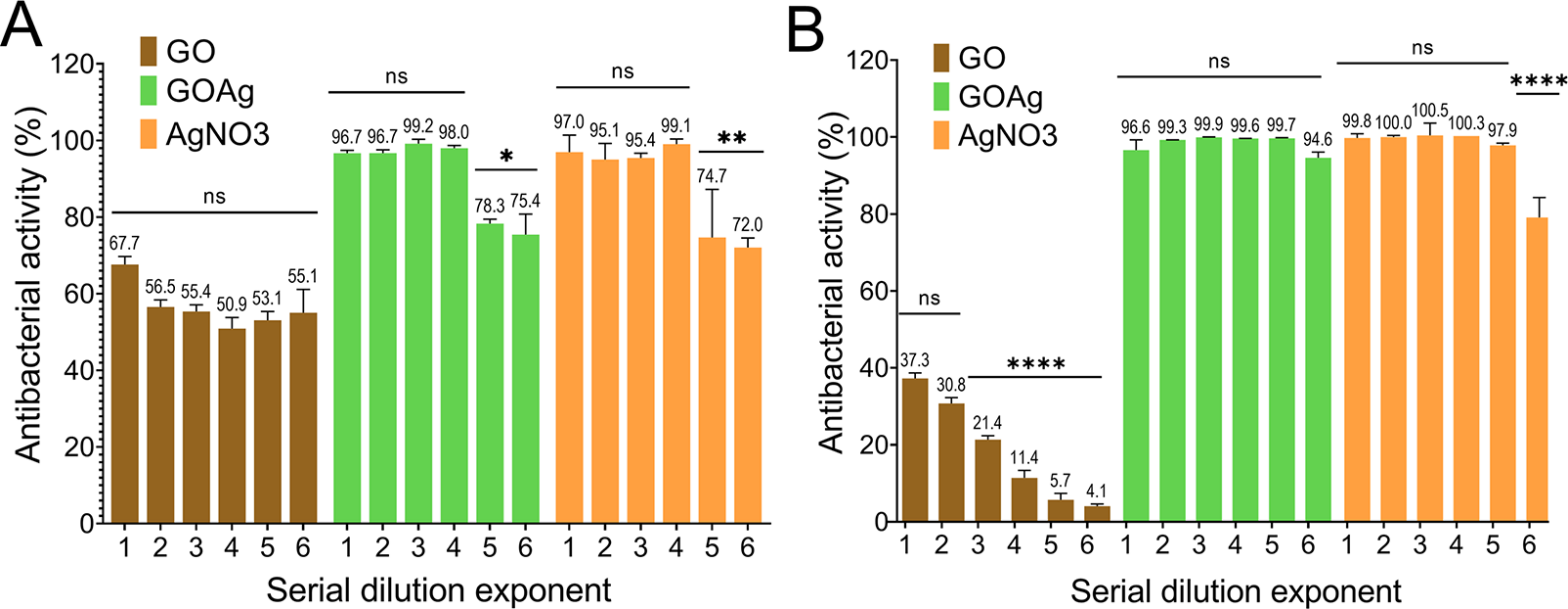
Antimicrobial activity of GO, GOAg, and AgNO_3_ diluted solutions to *S. aureus* (A) and *P. aeruginosa* (B).

The pristine GO sheets did not exhibit inhibitory action at the two base serial dilution concentrations tested. A previous study demonstrated that GOAg inhibited *S. aureus* cells at concentrations of released silver ions ranging from 30 to 60 μg mL^–1^ and *P. aeruginosa* from 15 to 60 μg mL^–1^.^38,60^ Our findings reveal that concentrations 35–70 μg mL^–1^ GOAg are enough to inactivate *S. aureus* cells, while *P. aeruginosa* is inhibited within the 17.5–70 μg mL^–1^ of released silver. Furthermore, the AgNO_3_ solution demonstrated strong bacteriostatic properties, exhibiting effectiveness at a minimum tested concentration of approximately 3.28 μg mL^–1^.

The BC-A-GOAg material demonstrated 93% effectiveness against *S. aureus* and 84% against *P. aeruginosa* , as shown in Figures 5A and 5B, in its ability to prevent adhesion and biofilm formation. Faria et al. found similar efficacy, ranging from 90% to 100%, against the same bacteria in cellulose acetate membranes incorporated with GOAg.^38^ In a direct assay, BC-A-GOAg exhibited approximately 74% antibacterial activity against *S. aureus* and 59% against *P. aeruginosa* (Figures 5C and 5D). These results are consistent with earlier observations by Faria et al., who reported a 79.4 ± 6.1% reduction in the activity of GOAg on PLGA-chitosan against *S*. *aureus*.^60^ However, the authors achieved better results against *P*. *aeruginosa*, with a 98% reduction.

**Figure 5:**
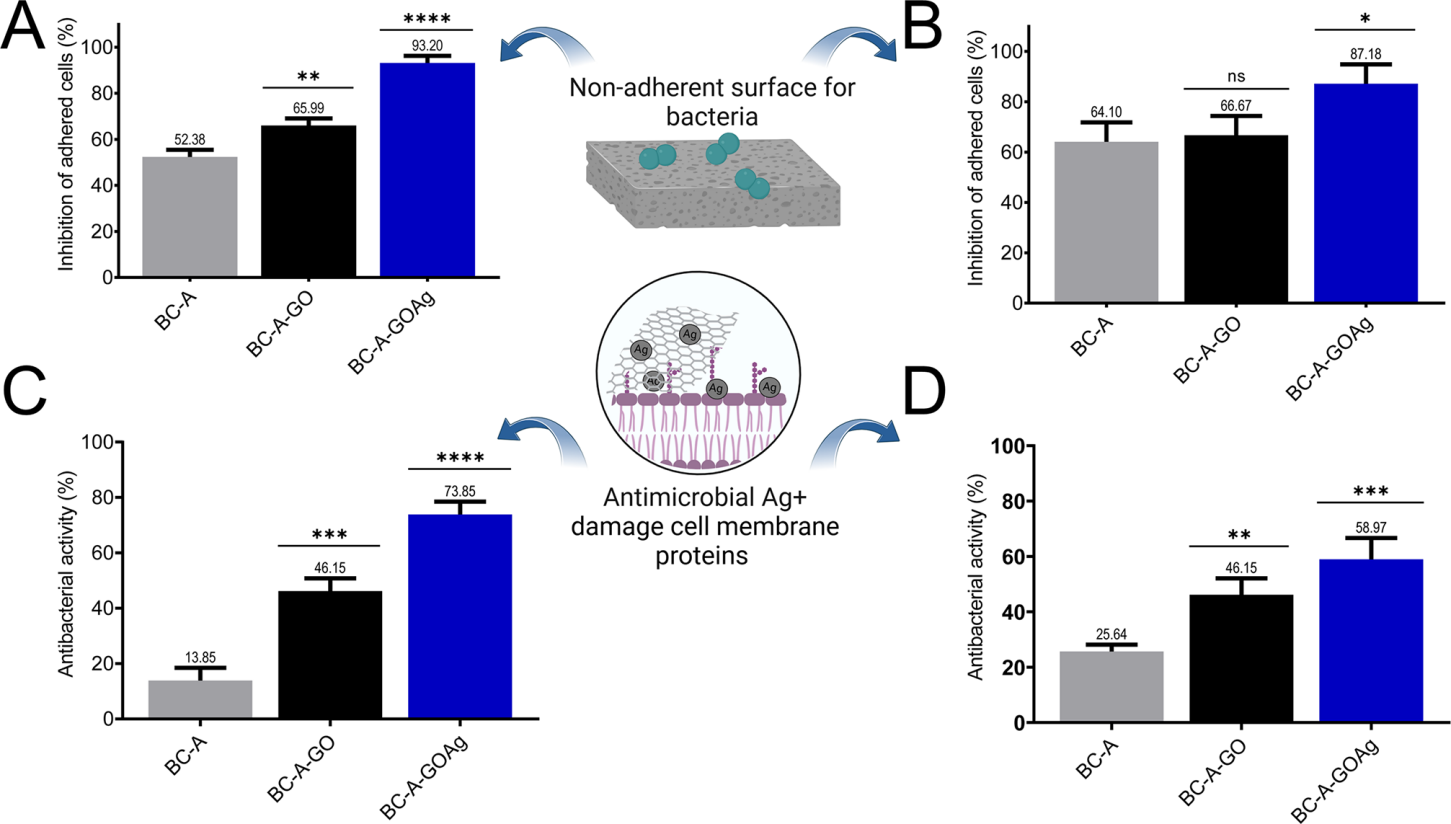
Antiadhesion activity of BC-A-GOAg compared to other membranes after 4 h of direct contact with a suspension of *S. aureus* (A) and *P. aeruginosa* (B). Antimicrobial activity of BC-A-GOAg compared to the other membranes in 4 h of direct contact with a suspension of *S. aureus* (C) and *P. aeruginosa* (D). Created in BioRender. Fonseca de Faria, A. (2025) https://BioRender.com/ifb7jb2.

In the context of indirect testing, the primary focus was on the bacterial activity against *S. aureus* resulting from silver released into a solution (PBS), as illustrated in Figures 6A and 6B. The antimicrobial results within the first 4 h of the assay indicate a 75% *S. aureus* inactivation under direct contact compared to 50% obtained from indirect contact. This suggests that direct interaction with the dressing surface significantly enhances bactericidal activity. Moreover, It was noted that the BC-A-GOAg dressing demonstrated antimicrobial properties comparable to those of *Aquacel Ag Extra* commercial dressings over a three-day period. This prolonged silver release is advantageous, as it allows for less frequent dressing changes, reducing unnecessary mechanical debridement during removal.^61^ When comparing the three types of membranes, it becomes evident that only a small quantity of silver was released in the first hours from BC-A-GOAg (Figure 3I), which primarily contributes to its antimicrobial activity. Additionally, the degradation products of the membranes in PBS released more Ag^+^ ions, further improving their antimicrobial properties.

**Figure 6:**
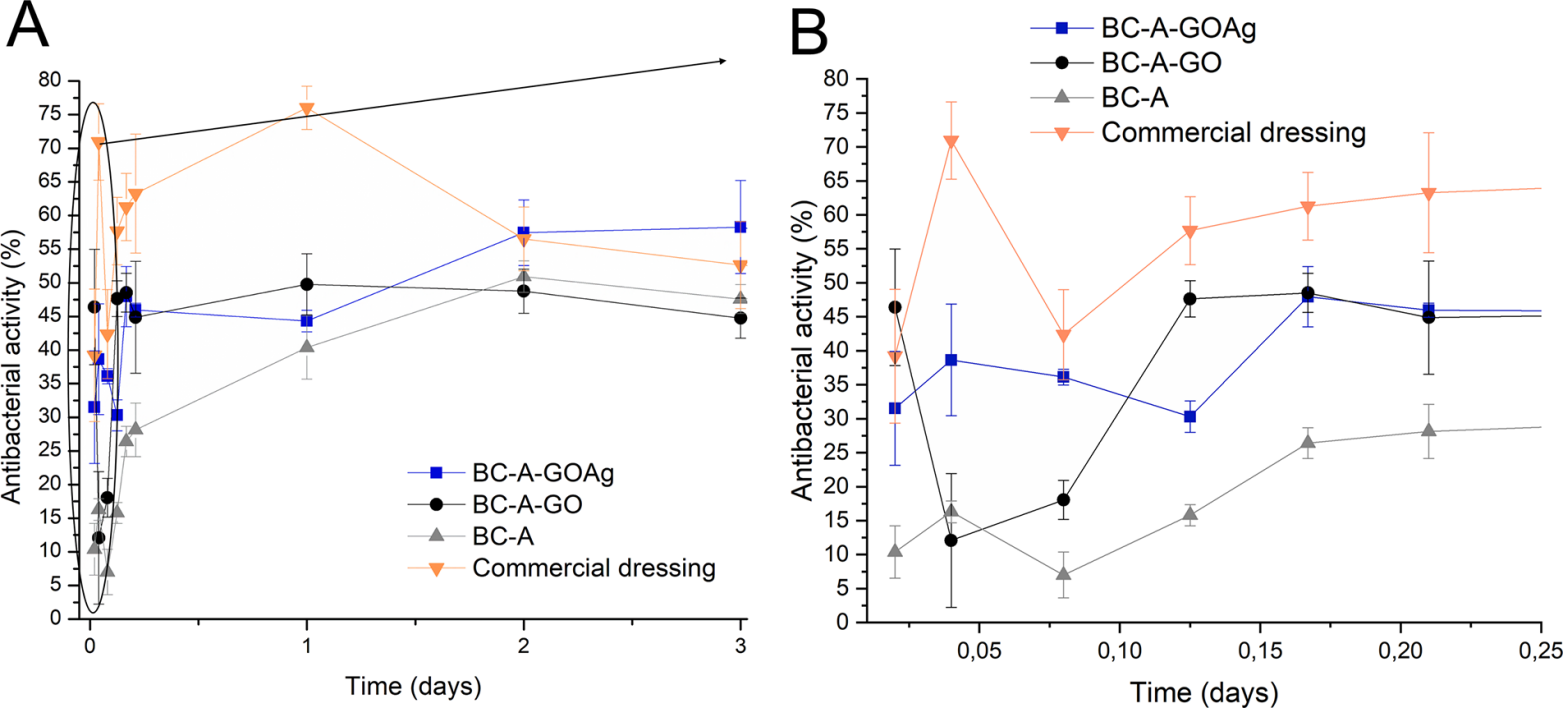
Antimicrobial activity of BC-A, BC-A-GO, and BC-A-GOAg membranes, extracted after 3 days of contact with PBS to release Ag^+^ ions, and commercial dressing membrane extracts against *S. aureus* (A) with approximation from 0 to 0.25 days (B).

### 3.3 *In* *Vitro* Cell Tests

The BC-A-GOAg extract demonstrated low cytotoxicity, with fibroblast viability of 57.03% at 24 h and 85.06% at 48 h (Figure 7). Although fibroblasts cultured in vitro showed reduced viability when exposed to the BC-A-GOAg extract, they demonstrated recovery within 48 h. In contrast, commercial dressings contain silver at concentrations approximately 30 times higher, which results in a cytotoxic effect on cells *in vitro* with no viability recovery, unlike the BC-A-GOAg extract.^62^ It was observed that increased concentrations of silver resulted in a more pronounced decrease in cell viability.^63^ This test indicates that isolated cells are notably highly sensitive to the effects of silver on the cell membrane. Future *in vivo* studies can further investigate the cytotoxic response in a complete organism.

**Figure 7:**
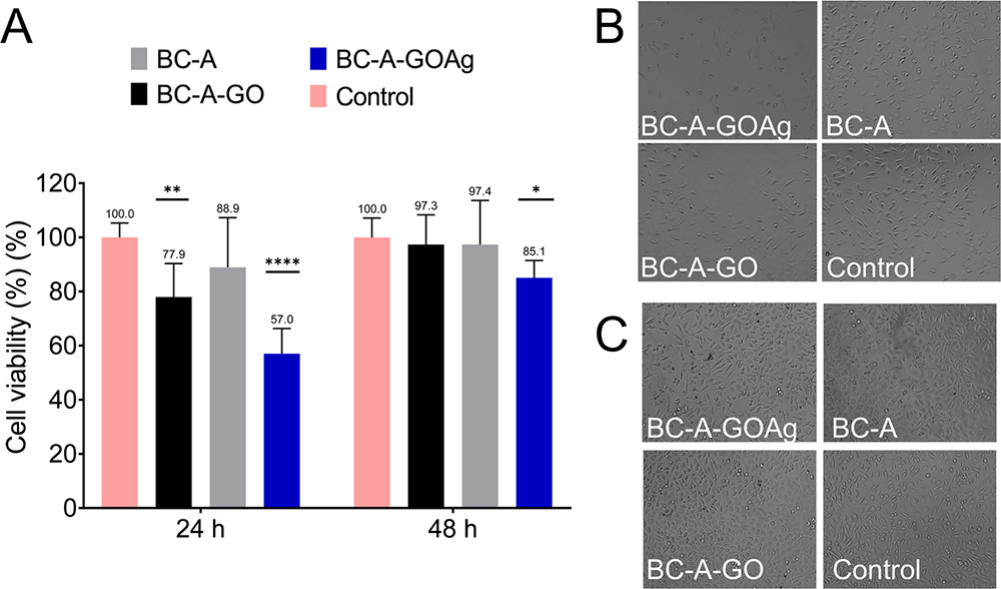
Cytotoxicity assay for BC-A, BC-A-GO, and BC-A-GOAg (A). Digital photographs of the BC-A, BC-A-GO, and BC-A-GOAg after 24 h (B) and 48 h (C) of contact with fibroblast cells. The control consisted of fibroblasts in supplemented DMEM.

BC-A-GOAg exhibited significant wound closure compared to the negative control, as shown in Figure 8A. The BC-A membrane achieved a notable percentage area difference of 29.4% at 18 h and 7% at 24 h in terms of scratch closure, as shown in Figure 8B. According to Choudhary et al., the addition of GOAg to membranes containing another polymer, such as chitosan, positively influenced wound closure outcomes in comparison to the control.^11^

**Figure 8:**
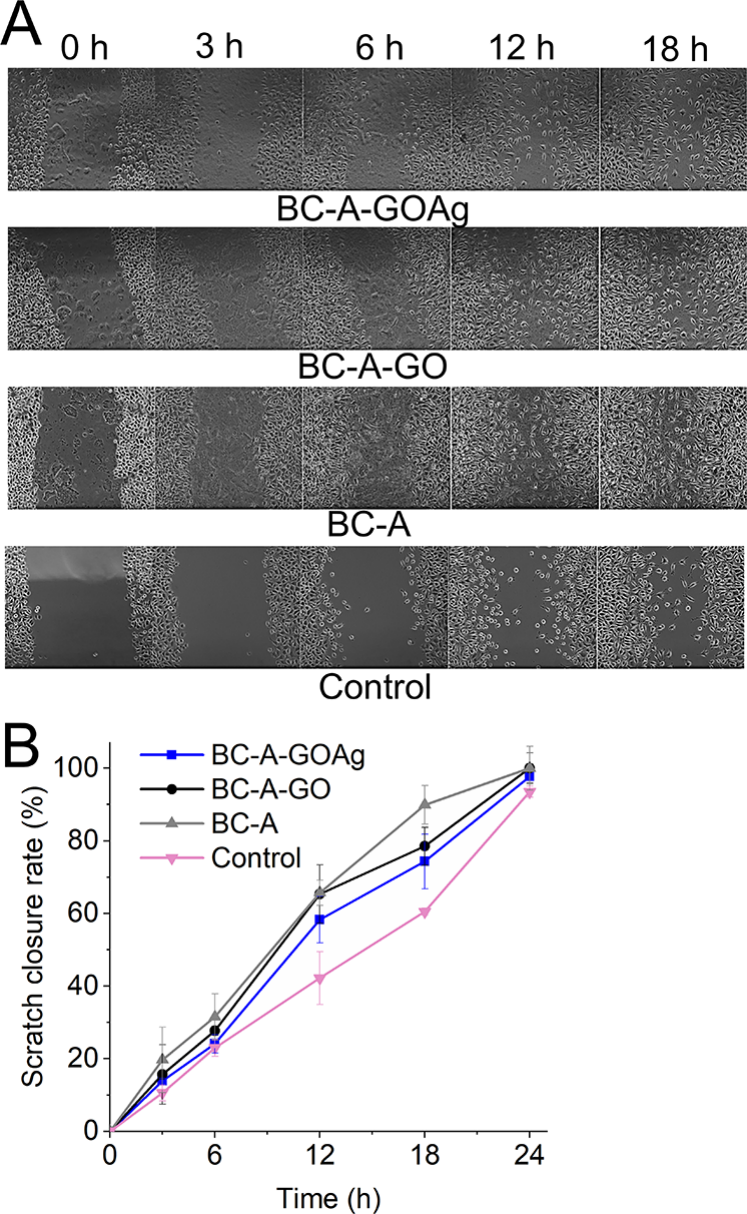
Digital photographs taken from the BC-A, BC-A-GO, and BC-A-GOAg as they underwent the scratch test with fibroblast cells (A). Scratch closure rate at 0, 3, 6, 12, and 24 h (B). The control group consisted of fibroblasts cultured in supplemented DMEM.

The inflammation test results were conducted using membrane extract (BC-A, BC-A-GO, and BC-A-GOAg) in contact with PBS over periods of 1 and 10 days. The absorbance was read at 650 nm with intervals ranging from 1 to 10 min, as described in the methodology (topic 2.2.8). These results are illustrated in Figure 9. As the duration of exposure increased, both BC-A and BC-A-GOAg samples showed promise in reducing inflammation. A lower absorbance value corresponds to a decreased inflammatory potential, reflecting the release of MPO by activated neutrophils. In contrast, the BC-A-GO sample exhibited minimal changes throughout the test, as the absorbance values remained practically stable, suggesting a lower anti-inflammatory potential of GO.

**Figure 9:**
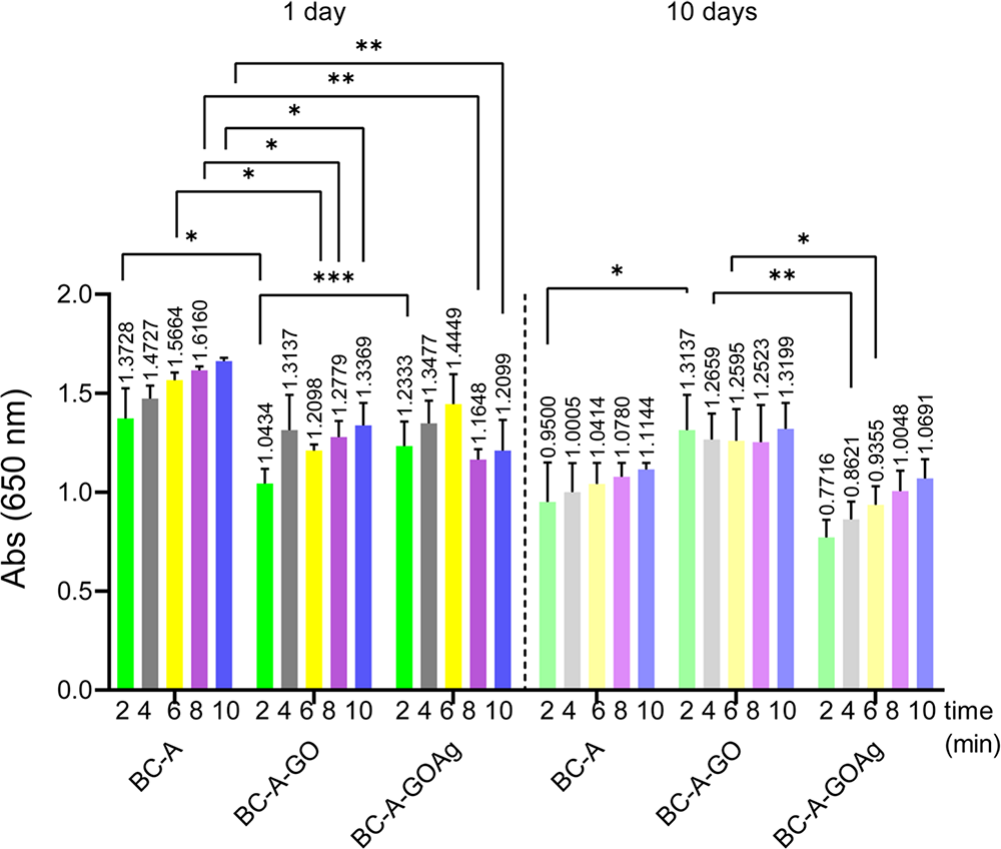
Inflammation assay performed to evaluate the release of MPO from neutrophils upon exposure to the BC-A, BC-A-GO, and BC-A-GOAg membrane extract over 1 and 10 day periods. Reading absorbances at 620 nm were conducted at 2, 4, 6, 8, and 10 min after a total 4 h interval of contact between the membrane extract and the cells.

In Figure 9, the samples were assessed individually over periods of 1 and 10 days. The variation in absorbance at 650 nm was measured during the first 5 min using the *Cayman* kit. The assessment included the kit’s positive reference control, which contains an MPO enzyme that triggers an inflammatory response, as well as a negative control, made of dressing samples containing an enzyme inhibitor to suppress any inflammatory reaction.

The results indicated that BC-A-GOAg has the potential to reduce inflammation. This potential is more evident in Figure 9, which evaluates the activity of the enzyme MPO per minute over the tested periods using eq 5. Figure 9 displays a significant decrease in the activity of the MPO enzyme in the BC-A-GOAg extract after 10 days, when compared to the activity observed at the 1-day mark.

The activity of the MPO enzyme in the inflammation test was measured after one and 10-day periods, as illustrated in Figures 10A and B. As shown in Figure 9, the BC-A-GOAg dressing exhibited a reduction in the release of enzymes from neutrophil granules. This was particularly evident in the enzyme activity observed in the 1-day extract test (Figure 10B) membrane compared to the 10-day extract samples. This suggests that the dressing may have a potential anti-inflammatory effect throughout its application.

**Figure 10:**
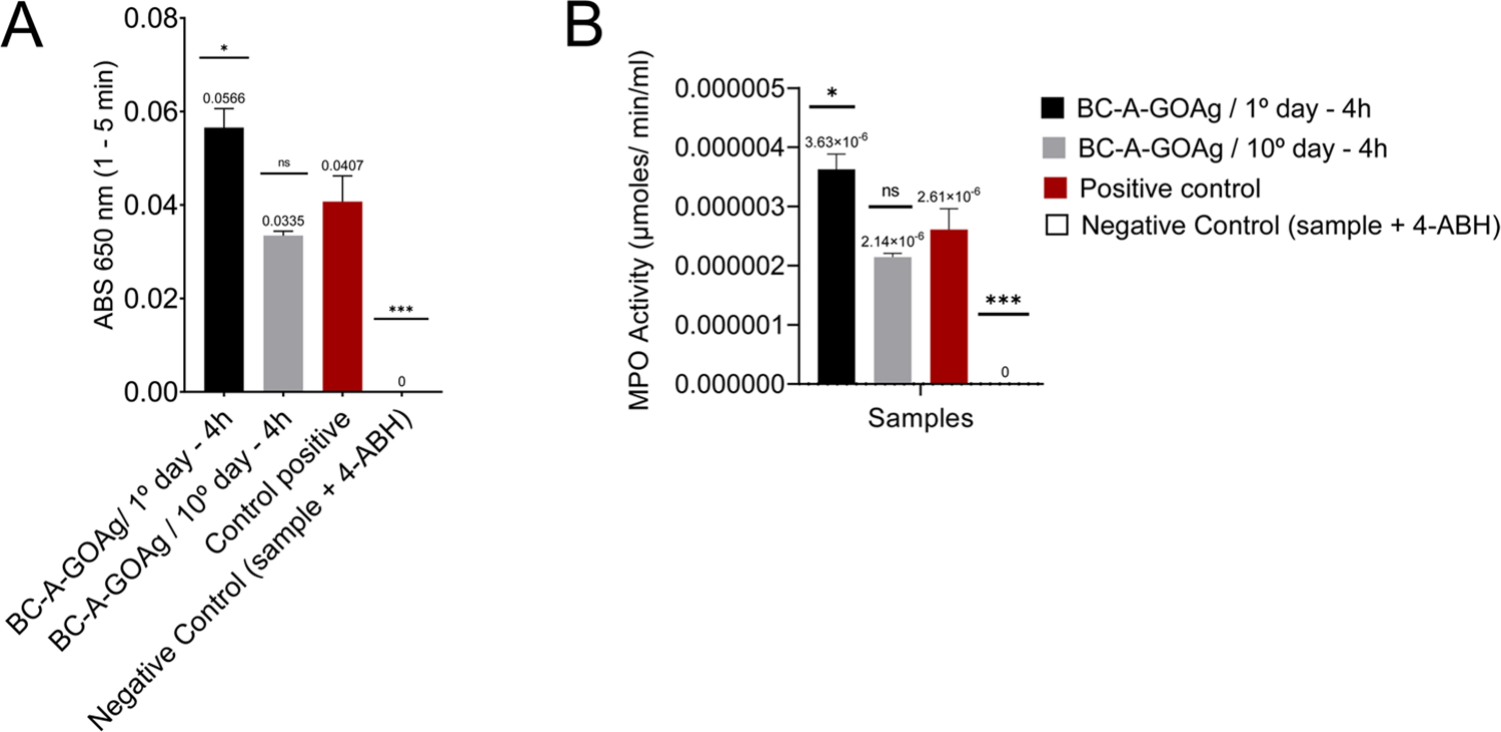
Inflammation test comparing between the BC-A-GOAg (first and 10th-day extracts), positive control (kit), negative control (sample + inhibitor kit), and GO and GOAg synthesis samples, in the 1–5 min range (A). Measurement of MPO enzyme activity (μmols/min/mL) against tests with BC-A-GOAg, positive and negative controls in the 4 h test interval (B).

Graphene has found numerous applications in health and medicine.^64,65^ In vivo tests conducted on male Wistar rats involved the implantation of chitosan-graphene (CS-GO) films to evaluate their biocompatibility in subdermal tissues over a 60-day period. The results showed that the film composed of graphene oxide demonstrated excellent biocompatibility, characterized by a low inflammatory response, effective healing, and notable tissue regeneration after the 60-day implantation period.^66^ However, the dressing sample that lacked silver exhibited a reduced anti-inflammatory potential when using isolated graphene. This potential was enhanced in the presence of silver, indicating a synergistic effect between silver and graphene oxide, which resulted in improved antimicrobial properties of BC-A-GOAg compared to BC-A-GO. These findings support the anti-inflammatory potential of graphene when combined with other compounds.

In this study, the properties of graphene combined with silver, forming BC-A-GOAg, were evaluated, resulting in a noticeable reduction in inflammation over time. Initially, the dressing extracts displayed inflammatory responses. Nevertheless, as time progressed, the pro-inflammatory potential diminished, likely due to the sustained release of silver and the initial activity of myeloperoxidases—an enzyme crucial for the bactericidal functions of the dressing in mammals, particularly in polymorphonuclear leukocytes and neutrophils, which are directly involved in O_2_-dependent bactericidal mechanisms.^67^ Consequently, this initial enhancement, followed by a decrease in inflammation, proves beneficial, as it is vital for preventing infections and promoting efficient wound healing.

## 4 Conclusions

The BC-A, BC-A-GO, and BC-A-GOAg membranes exhibited large, overlapping pores that enhanced water absorption. Direct and indirect antibacterial i*n vitro* studies revealed that GO-Ag nanoparticles contributed to the membranes’ antimicrobial efficacy. Analysis of silver release indicated a mechanism through which the released silver maintained continuous and effective antimicrobial activity, achieving effectiveness of up to 93% against S. aureus and 84% against *P. aeruginosa*, thereby preventing bacterial adhesion. The findings suggest that BC-A-GOAg exhibited no cytotoxicity within 48 h. Additionally, the reduction in myeloperoxidase enzyme activity may indicate potential anti-inflammatory properties of the functionalized membranes. Given these promising results, future studies should focus on evaluating the dressing’s effectiveness in vivo models for a more comprehensive understanding of its properties and benefits in wound healing processes.

## Supplementary Material



## Notes

Raman spectra for GO and GOAg nanomaterials (Figure S1) showing D, G, and 2D bands characteristic of GO and the Ag–O peaks of silver (PDF)

## References

[1] Cavallo I., Sivori F., Mastrofrancesco A., Abril E., Pontone M., Di Domenico E. G., Pimpinelli F. (2024). Bacterial Biofilm in Chronic Wounds
and Possible Therapeutic Approaches. Biology.

[2] Sankar S., Kodiveri Muthukaliannan G. (2024). Deciphering
the Crosstalk between
Inflammation and Biofilm in Chronic Wound Healing: Phytocompounds
Loaded Bionanomaterials as Therapeutics. Saudi
J. Biol. Sci..

[3] Woo K. (2024). The Chronic
Wound-Related Pain Model: Holistic Assessment and Person-Centered
Treatment. Clin Geriatr Med..

[4] Raffetto J. D., Ligi D., Maniscalco R., Khalil R. A., Mannello F. (2020). Why Venous
Leg Ulcers Have Difficulty Healing: Overview on Pathophysiology, Clinical
Consequences, and Treatment. J. Clin. Med..

[5] Gardikiotis I., Cojocaru F. D., Mihai C. T., Balan V., Dodi G. (2022). Borrowing
the Features of Biopolymers for Emerging Wound Healing Dressings:
A Review. International Journal of Molecular
Sciences.

[6] Scotton M. F., Miot H. A., Abbade L. P. F. (2014). Factors
That Influence Healing of
Chronic Venous Leg Ulcers: A Retrospective Cohort. An Bras Dermatol.

[7] Ye J., Li J., Wang X., Wang Q., Wang S., Wang H., Zhu H., Xu J. (2024). Preparation of Bacterial Cellulose-Based Antibacterial
Membranes with Prolonged Release of Drugs: Emphasis on the Chemical
Structure of Drugs. Carbohydr. Polym..

[8] Sun M., Li D., Xi Y., Qin X., Liao Y., Liu X., Jia S., Xie Y., Zhong C. (2024). NIR-Triggered Bacterial Cellulose-Based
Wound Dressings for Multiple Synergistic Therapy of Infected Wound. Int. J. Biol. Macromol..

[9] Zhang W., Zhao S., Guan Q., Li P., Fan Y. (2024). Enhancing
Chronic Wound Healing through Engineering Mg2+-Coordinated Asiatic
Acid/Bacterial Cellulose Hybrid Hydrogels. ACS
Appl. Mater. Interfaces.

[10] Monteiro R. T., Da Silva T. F., Filho Raimundo N. F.
M., Vasconcelos N. F., Nogueira Karina A. B., Petrilli R., Andrade F. K., Vieira R. S. (2023). Simvastatin-Loaded
Alginate Bilayer Membranes for Wound Dressing Applications. J. Drug Deliv Sci. Technol..

[11] Hu H., Xu F. J. (2020). Rational Design and Latest Advances of Polysaccharide-Based
Hydrogels
for Wound Healing. Biomaterials Science.

[12] Moreira
Filho R. N. F., Vasconcelos N. F., Andrade F. K., Rosa M. de F., Vieira R. S. (2020). Papain Immobilized on Alginate Membrane for Wound Dressing
Application. Colloids Surf. B Biointerfaces.

[13] Sun Z., Hu K., Wang T., Chen X., Meng N., Peng X., Ma L., Tian D., Xiong S., Zhou C., Yang Y. (2024). Enhanced Physiochemical,
Antibacterial, and Hemostatic Performance of Collagen-Quaternized
Chitosan-Graphene Oxide Sponges for Promoting Infectious Wound Healing. Int. J. Biol. Macromol..

[14] Elhami N., Pazhang M., Beygi-khosrowshahi Y., Dehghani A. (2024). Development of Nanocomposites
Based on Chitosan/Reduced Graphene Oxide for Wound Healing Application. Int. J. Biol. Macromol..

[15] Ningrum D. R., Hanif W., Mardhian D. F., Asri L. A. T. W. (2023). In Vitro Biocompatibility
of Hydrogel Polyvinyl Alcohol/Moringa Oleifera Leaf Extract/Graphene
Oxide for Wound Dressing. Polymers (Basel).

[16] Qureshi M. A. ur R., Arshad N., Rasool A. (2023). Graphene Oxide
Reinforced Biopolymeric
(Chitosan) Hydrogels for Controlled Cephradine Release. Int. J. Biol. Macromol..

[17] Xu W. P., Zhang L. C., Li J. P., Lu Y., Li H. H., Ma Y. N., Wang W. Di, Yu S. H. (2011). Facile
Synthesis
of Silver@graphene Oxide Nanocomposites and Their Enhanced Antibacterial
Properties. J. Mater. Chem..

[18] de
Souza Bernardes M., de Sales J. D. L., Medeiros Borsagli F. G. L. (2025). Antioxidant
and Antimicrobial Activity of Innovative Carboxymethyl Cellulose/Graphene
Oxide Nanocomposite for Biological Proposals. J. Mol. Liq..

[19] Prasad C., Madkhali N., Lee B. M., Kang C. S., Choi H. Y. (2023). Recent
Developments in GO/Cellulose Based Composites: Properties, Synthesis,
and Its Applications. Polymer (Guildf).

[20] Phong M. T., Nguyen T. A., Nguyen
Thi Yen N., Tran V. K., Vuong V. D., Nguyen M. H., Pham T. T., Le T. V. (2024). Evaluation of Green-Synthesized
Silver Nanoparticle-Loaded Graphene Oxide (AgNPs@GO) Nanocomposite
toward Bio Logical Wastewater Filtration. Case
Stud. Chem. Environ. Eng..

[21] Hosseini S. N., Jalaly M., Heydari M., Mirzapoor A. (2024). Evaluation
of a Chitosan-Based Hydrogel Containing Graphene Oxide and Scrophularia
Striata Extract as an Antimicrobial Wound Dressing. South African Journal of Botany.

[22] Yang S., Zhang C., Yong L., Niu M., Cheng W., Zhang L., Xue B. (2024). Construction of PNIPAM/Graphene
Oxide
Loaded with Silver Nanoparticles Interpenetrating Intelligent Hydrogels
for Antibacterial Dressing. Polym. Bull..

[23] Durairaj S., Sridhar D., Ströhle G., Li H., Chen A. (2024). Bactericidal
Effect and Cytotoxicity of Graphene Oxide/Silver Nanocomposites. ACS Appl. Mater. Interfaces.

[24] Pal S., Nisi R., Stoppa M., Licciulli A. (2017). Silver-Functionalized
Bacterial Cellulose as Antibacterial Membrane for Wound-Healing Applications. ACS Omega.

[25] Scott C., Wisdom N. H., Coulter K., Bardin S., Strap J. L., Trevani L. (2023). Interdisciplinary Undergraduate
Laboratory for an Integrated
Chemistry/Biology Program: Synthesis of Silver Nanoparticles (AgNPs)-Cellulose
Composite Materials with Antimicrobial Activity. J. Chem. Educ..

[26] Munhoz L. L. S., Alves M. T. O., Alves B. C., Nascimento M. G. F. S., Sábio R. M., Manieri K. F., Barud H. S., Esquisatto M. A. M., Aro A. A., de Roch Casagrande L., Silveira P. C. L., Santos G. M. T., Andrade T. A. M., Caetano G. F. (2023). Bacterial
Cellulose Membrane Incorporated with Silver Nanoparticles for Wound
Healing in Animal Model. Biochem. Biophys. Res.
Commun..

[27] Luz E. P. C. G., da Silva T. F., Marques L. S. M., Andrade A., Lorevice M. V. V., Andrade F. K., Yang L., de Souza Filho A. G., Faria A. F., Silveira Vieira R. (2024). Bacteria-Derived Cellulose Membranes
Modified with Graphene Oxide-Silver Nanoparticles for Accelerating
Wound Healing. ACS Appl. Bio Mater..

[28] Turkevich J., Stevenson P. C., Hillier J. (1951). A Study of the Nucleation and Growth
Processes in the Synthesis of Colloidal Gold. Discuss. Faraday Soc..

[29] de
Moraes A. C. M., Lima B. A., de Faria A. F., Brocchi M., Alves O. L. (2015). Graphene Oxide-Silver Nanocomposite as a Promising
Biocidal Agent against Methicillin-Resistant Staphylococcus Aureus. Int. J. Nanomedicine.

[30] HESTRIN S., SCHRAMM M. (1954). Synthesis of Cellulose
by Acetobacter Xylinum. II.
Preparation of Freeze-Dried Cells Capable of Polymerizing Glucose
to Cellulose. Biochem. J..

[31] Vasconcelos N. F., Andrade F. K., Vieira L. de A. P., Vieira R. S., Vaz J. M., Chevallier P., Mantovani D., Borges M. de F., Rosa M. de F. (2020). Oxidized
Bacterial Cellulose Membrane as Support for Enzyme Immobilization:
Properties and Morphological Features. Cellulose.

[32] Kirdponpattara S., Khamkeaw A., Sanchavanakit N., Pavasant P., Phisalaphong M. (2015). Structural
Modification and Characterization of Bacterial Cellulose-Alginate
Composite Scaffolds for Tissue Engineering. Carbohydr. Polym..

[33] Franz T. J. (1975). PERCUTANEOUS
ABSORPTION ON THE RELEVANCE OF IN VITRO DATA. J. Investigative Dermatol..

[34] United States Pharmacopeial Convention. SEMISOLID DRUG—PERFORMANCE. Chapter 1724; 2014. http://www.fda.gov/downloads/.

[35] Liu Y. L., Su Y. H., Lee K. R., Lai J. Y. (2005). Crosslinked Organic–Inorganic
Hybrid Chitosan Membranes for Pervaporation Dehydration of Isopropanol–Water
Mixtures with a Long-Term Stability. J. Membr.
Sci..

[36] ASTM. ASTM D638–99. Standard Test Method for Tensile Properties of Plastics; West Conshohocken: PA, USA, 1999. www.astm.org.

[37] Clinical and Laboratory Standards Institute. M07-A10: Methods for Dilution Antimicrobial Susceptibility Tests for Bacteria That Grow Aerobically; Approved Standard—Tenth Edition; 2015; pp 15–47. www.clsi.org.

[38] de
Faria A. F., de Moraes A. C. M., Andrade P. F., da Silva D. S., do Carmo Gonçalves M., Alves O. L. (2017). Cellulose Acetate
Membrane Embedded with Graphene Oxide-Silver Nanocomposites and Its
Ability to Suppress Microbial Proliferation. Cellulose.

[39] Monteiro R. T., Da Silva T. F., de Souza Guedes L., Moreira Filho R. N. F., Soares A. L. B., Vasconcelos N. F., Andrade F. K., Vieira R. S. (2024). Porous
and Dense Alginate/Chitosan Composite Films Loaded with Simvastatin
for Dressing Applications. Coatings.

[40] INTERNATIONAL STANDARD. ISO 10993–5. Biological Evaluation of Medical — Part 5: Tests for in Vitro Cytotoxicity; Switzerland, 2009.

[41] INTERNATIONAL STANDARD. ISO 10993–12. Biological Evaluation of Medical Devices — Part 12: Sample Preparation and Reference Materials; Switzerland, 2012. www.iso.org.

[42] da
Silva T. F., Leite T. A., de Souza F. F. P., da
Silva Barroso W., de Souza Guedes L., da Silva A. L. C., de
Souza B. W. S., Vieira R. S., Andrade F. K. (2024). Loading of Bacterial
Cellulose Dressing with Frutalin, a Lectin from Artocarpus Incisa
L. Int. J. Biol. Macromol..

[43] Schneider C. A., Rasband W. S., Eliceiri K. W. (2012). NIH Image
to ImageJ: 25 Years of
Image Analysis. Nat. Methods.

[44] Taymour N., Hussein Abdel Kader S., Aboushelib M. N., Gad M. M. (2024). Comparative Analysis
of Dimensional Changes in Autoclavable Polyvinyl Siloxane (PVS) Impressions
under Various Sterilization/Disinfection Protocols: A Randomized Controlled
Trial. Saudi Dental Journal.

[45] Soares J. d. P., Dos Santos J. E., Chierice G. O., Cavalheiro É.
T. G. (2004). Thermal Behavior
of Alginic Acid and Its Sodium Salt. Ecl. Quím.

[46] Teixeira S. R. Z., Dos Reis E. M., Apati G. P., Meier M. M., Nogueira A. L., Formolo Garcia M. C., Dos Santos Schneider A. L., Testa
Pezzin A. P., Porto L. M. (2019). Biosynthesis and Functionalization
of Bacterial Cellulose Membranes with Cerium Nitrate and Silver Nanoparticles. Mater. Res..

[47] Andrade F. K., Morais J. P. S., Muniz C. R., Nascimento J. H. O., Vieira R. S., Gama F. M. P., Rosa M. F. (2019). Stable
Microfluidized
Bacterial Cellulose Suspension. Cellulose.

[48] Lama G. C., Santillo C., Recupido F., Liu J., Verdolotti L., Marzella R., Polichetti T., Kaciulis S., Lavorgna M. (2024). Autoclave-Mediated
Reduction of Graphene Oxide for Enhanced Conductive Films. Appl. Surf. Sci..

[49] Jiang Y., Yu G., Zhou Y., Liu Y., Feng Y., Li J. (2020). Effects of
Sodium Alginate on Microstructural and Properties of Bacterial Cellulose
Nanocrystal Stabilized Emulsions. Colloids Surf.
A Physicochem Eng. Asp.

[50] Moreira
Filho R. N. F., Vasconcelos N. F., Andrade F. K., Rosa M. de F., Vieira R. S. (2020). Papain Immobilized on Alginate Membrane for Wound Dressing
Application. Colloids Surf. B Biointerfaces.

[51] Aichour A., Zaghouane-Boudiaf H. (2020). Single and Competitive Adsorption Studies of Two Cationic
Dyes from Aqueous Mediums onto Cellulose-Based Modified Citrus Peels/Calcium
Alginate Composite. Int. J. Biol. Macromol..

[52] Forouzandehdel S., Meskini M., Rami M. R. (2020). Design
and Application of (Fe3O4)-GOTfOH
Based AgNPs Doped Starch/PEG-Poly (Acrylic Acid) Nanocomposite as
the Magnetic Nanocatalyst and the Wound Dress. J. Mol. Struct..

[53] Torabi S., Nasiriani T., Javanbakht S., Shaabani A. (2025). Anchoring Silver Nanoparticles
on Graphene Quantum Dots: A Highly Efficient, Green, and Rapid Nano-Catalyst
for the Reduction of Nitro Compounds and Tandem Reductive Ugi Reactions. J. Phys. Chem. Solids.

[54] Da
Silva Pereira B., Silva M. F., Bittencourt P. R. S., De Oliveira D. M. F., Pineda E. A. G., Hechenleitner A. A. W. (2015). Cellophane
and Filter Paper as Cellulosic Support for Silver Nanoparticles and
Its Thermal Decomposition Catalysis. Carbohydr.
Polym..

[55] Zhang Q., Ma F., Tan W., Liu L., Jing M., Sun T. (2023). Enhanced Heat
Storage Performance of CaCl2·6H2O Using BN Nanosheet as an Additive. Heat and Mass Transfer/Waerme- und Stoffuebertragung.

[56] Li Y., Li Y., Li C., Zhang X., Zeng F., Lin H., Su Z. (2020). Optical and
Mechanical Properties of NaCl: Ce3+ Crystal Grown by
the Czochralski Method. Journal of Materials
Science: Materials in Electronics.

[57] Vasconcelos N. F., Chevallier P., Mantovani D., Rosa M. de F., Barros F. J. S., Andrade F. K., Vieira R. S. (2024). Oxidized Bacterial Cellulose Membranes
Immobilized with Papain for Dressing Applications: Physicochemical
and In Vitro Biological Properties. Pharmaceutics.

[58] Moser H., Rodrigues Pereima R., Lopes Pereima M. J., Maurício C., Lopes J., Rua P., Barbosa R. (2013). Evolução
Dos Curativos de Prata No Tratamento de Queimaduras de Espessura Parcial
Evolution of Silver Dressings in the Treatment of Partial Thickness
Burns. Rev. Bras Queimaduras.

[59] Shahriari-Khalaji M., Hong S., Hu G., Ji Y., Hong F. F. (2020). Bacterial
Nanocellulose-Enhanced Alginate Double-Network Hydrogels Cross-Linked
with Six Metal Cations for Antibacterial Wound Dressing. Polymers.

[60] De
Faria A. F., Perreault F., Shaulsky E., Arias Chavez L. H., Elimelech M. (2015). Antimicrobial Electrospun Biopolymer Nanofiber Mats
Functionalized with Graphene Oxide-Silver Nanocomposites. ACS Appl. Mater. Interfaces.

[61] Gokaltun A. A., Fan L., Mazzaferro L., Byrne D., Yarmush M. L., Dai T., Asatekin A., Usta O. B. (2023). Supramolecular Hybrid Hydrogels as
Rapidly On-Demand Dissoluble, Self-Healing, and Biocompatible Burn
Dressings. Bioact Mater..

[62] Bourdillon K. A., Delury C. P., Cullen B. M. (2017). Biofilms
and Delayed Healing –
an in Vitro Evaluation of Silver- and Iodine-Containing Dressings
and Their Effect on Bacterial and Human Cells. Int. Wound J..

[63] El-Sapagh S. H., El-Zawawy N. A., Elshobary M. E., Alquraishi M., Zabed H. M., Nouh H. S. (2024). Harnessing
the Power of Neobacillus
Niacini AUMC-B524 for Silver Oxide Nanoparticle Synthesis: Optimization,
Characterization, and Bioactivity Exploration. Microb Cell Fact.

[64] Mbayachi V. B., Ndayiragije E., Sammani T., Taj S., Mbuta E. R., khan A. U. (2021). Graphene Synthesis, Characterization
and Its Applications:
A Review. Results Chem..

[65] Urade A. R., Lahiri I., Suresh K. S. (2023). Graphene Properties. Synthesis and Applications: A Review. Jom.

[66] Valencia A. M., Valencia C. H., Zuluaga F., Grande-Tovar C. D. (2021). Synthesis
and Fabrication of Films Including Graphene Oxide Functionalized with
Chitosan for Regenerative Medicine Applications. Heliyon.

[67] Pereira C. C., Fonseca L. F. L. d., Santos M. V. d., Rodrigues P. H. M., Borelli P. (2000). Avaliação Da Atividade Da Mieloperoxidase
Neutrofílica Em Bovinos Da Raça Holandesa e Sua Correlação
Com Níveis Plasmáticos de Ácido Ascórbico. Revista Brasileira de Ciência Veterinária.

