# Supplementary material for: Advanced Foam Dressing of Modified Graphene Nanoparticles Loaded in Bacterial Cellulose/Calcium Alginate Matrix
Source: ACS Omega. 2025 Jun 30;10(27):28969–81. doi: 10.1021/acsomega.5c00609 (PMC12268464; doi:10.1021/acsomega.5c00609)
Supplement: Supplementary file 1 [file ao5c00609_si_001.pdf]

SUPPORTING INFORMATION

Advanced foam dressing of modified graphene nanoparticles loaded in bacterial cellulose/calcium alginate matrix.

Thamyres Freire da Silva[a], *Jacilane Ximenes Mesquita*[a], *Erika Patricia Chagas Gomes Luz*[a], *Alexandre Lopes Andrade*[b], *Henry Kobs*[d], *Edson Holanda Teixeira*[b], *Antônio Gomes de Souza Filho*[c], *Andreia Fonseca de Faria*[d], *Adriano Lincoln Albuquerque Mattos*[e], *Fábia Karine Andrade*[a] *and Rodrigo Silveira Vieira* [a]*.

[a] Adsorption Separations Research Group, Department of Chemical Engineering, Federal University of Ceará, 60455-760, Fortaleza – Ceará, Brazil

[b] Integrated Biomolecules Laboratory, Department of Pathology and Forensic Medicine, Federal University of Ceará, 60430350, Fortaleza- Ceará, Brazil

[c] Department of Physics, Federal University of Ceará, 60440-900, Fortaleza - Ceará, Brazil

[d] Department of Environmental Engineering Sciences, University of Florida, 32611, Gainesville - Florida, USA

[e] Embrapa Tropical Agroindustry, 60511-110, Fortaleza, Ceará, Brazil

SECTION 1: SUPPLEMENTARY FIGURES & TABLES

Figure S1. Figure showing the Raman spectra for pristine GO and GO-Ag materials. Page S3

SECTION 1: SUPPLEMENTARY FIGURES & TABLES

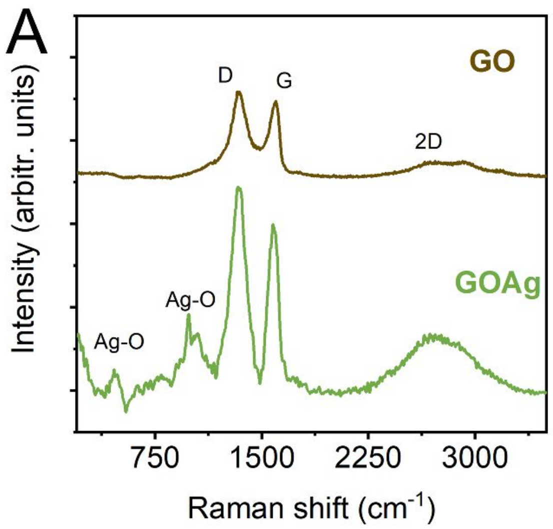

Figure S1: Raman spectra of GO and GOAg nanomaterials.
